# Sex Differences in Cardiovascular Disease Risk Factor Prevalence, Morbidity, and Mortality in Colombia: Findings from the Prospective Urban Rural Epidemiology (PURE) Study

**DOI:** 10.5334/gh.1289

**Published:** 2024-01-24

**Authors:** Jose Patricio Lopez-Lopez, Martin Rebolledo-Del Toro, Daniel Martinez-Bello, Ángel A Garcia-Peña, Gary O’Donovan, Maritza Perez-Mayorga, Johanna Otero, Sumathy Rangarajan, Salim Yusuf, Patricio Lopez-Jaramillo

**Affiliations:** 1 Masira Research Institute, Universidad de Santander (UDES), Bucaramanga, Colombia; 2 Internal Medicine Department, Cardiology Unit, Hospital Universitario San Ignacio, Pontificia Universidad Javeriana, Bogotá, Colombia; 3 School of Medicine, Universidad Militar Nueva Granada, Clínica Marly, Bogotá, Colombia; 4 The Population Health Research Institute, McMaster University, Hamilton, Canada; 5 Department of Medicine, McMaster University, Hamilton, Canada; 6 Universidad UTE, Facultad de Ciencias de la Salud Eugenio Espejo, Quito, Ecuador

**Keywords:** Gender, risk factors, Cardiovascular disease

## Abstract

**Background:**

Controversies exist on whether the presence of cardiovascular risk factors and their association with major cardiovascular events (MACE) is different between men and women. Most of the evidence comes from high-income countries, hindering extrapolation of sociocultural and demographic factors of other regions.

**Objective:**

To evaluate sex differences in the prevalence of cardiovascular risk factors and the incidence of MACE and diabetes in Colombian adults.

**Methods:**

We performed a survival analysis from women and men aged 35–70 belonging to the Prospective Urban Rural Epidemiology-Colombia prospective study. Incidence rates for MACE composite (myocardial infarction, stroke, heart failure, death) and each outcome and diabetes were calculated. Kaplan-Meier curves and log-rank tests were performed. The association between demographic, behavioral, and metabolic variables with MACE and diabetes were evaluated with Cox proportional hazards models.

**Results:**

7,552 participants (50±9.7 years) were included; 64% were women. Women had higher hypertension prevalence, body mass index, levels of total cholesterol, LDL-c, and HDL-c but lower triglycerides levels. Women were more sedentary but fewer smokers or active alcohol consumers and had higher educational levels. After 12-year mean follow-up (SD 2.3), the incidence rate of MACE composite was higher in men [4.2 (3.6–4.9) vs. 3.2 (2.8–3.7) cases per 1000 person-years]. Diabetes had the greatest association with MACE (HR = 2.63 95%CI:1.85;3.76), followed by hypertension (HR = 1.75 95%CI:1.30;2.35), low relative grip strength (HR = 1.53 95%CI:1.15;2.02), smoking (HR = 1.47 95%CI: 1.11;1.93), low physical activity (HR = 1.42 95%CI: 1.03;1.96). When evaluating risk factors by sex, only an increased waist-to-hip ratio was more strongly associated with MACE in men (p-interaction <0.05).

**Conclusions:**

The composite MACE outcome was higher in men despite having a lower overall burden of risk factors. The risk factors contribution was similar, leading us to reconsider the need to carrying out differentiated cardiovascular risk prevention and management campaigns, at least in our region.

## 1. Introduction

Cardiovascular disease (CVD) is the leading cause of morbidity and mortality in Colombia and elsewhere in Latin America [1]. In addition, over 70% of CVD can be attributed to a cluster of modifiable risk factors [2]. However, whether the contribution of these risk factors is different between men and women is still controversial. Historically, CVD, particularly coronary heart disease, has been reported to be more frequent in men and treated more aggressively [3,4]. Although recent studies showed that the prevalence of risk factors, incidence of major cardiovascular events (MACE), and cardiovascular death are similar between both sexes, others reported a higher prevalence and incidence in women [5,6]. Sexual disparities in cardiovascular risk and occurrence of MACE arise from a multifaceted interplay of genetic, hormonal, and environmental factors [7]. For example, in comparison to men, women have a higher likelihood of presenting with nonobstructive coronary artery disease and coronary microvascular dysfunction [8]. While the decline in estrogen levels in women has been linked to an increased cardiovascular risk, particularly due to elevated cholesterol levels, a controversial aspect arises from the lack of discernible benefits demonstrated in large clinical trials evaluating estrogen’s potential cardioprotective effects [9,10]. Accordingly, a range of initiatives have emerged to bolster women’s awareness and empowerment regarding their cardiovascular health. Furthermore, clinical practice guidelines have been established, outlining gender-specific strategies for the effective control and management of cardiovascular risks. [11,12]. Nevertheless, most of the data supporting these initiatives comes from high-income countries (HICs) [11,13,14,15], making it difficult to extrapolate typical sociocultural and demographic factors of other regions of the world. Moreover, the heaviest burden of CVD is reported on a global scale within low- and middle-income countries (LMICs), and the attributes of metabolic, behavioral, and sociodemographic risk factors in these regions diverge from those in HICs [2,16]. The Prospective Urban Rural Epidemiology (PURE) study evaluated 202,072 individuals from 27 HICs and LMCIs, reporting a lower burden of cardiovascular risk factors and the incidence of CVD in women [17]. Due to these discrepancies in the reports in the literature, possibly explained by the characteristics of the studied populations and different methodologies employed, the objective of the present analysis was to evaluate sex differences in the prevalence of cardiovascular risk factors and the incidence of MACE and diabetes in Colombian adults.

## 2. Methods

### 2.1 Study design and participants

The PURE study methodology has been described in detail previously [18]. We performed a survival analysis on the prospective PURE-Colombia cohort. In the baseline enrollment phase, participants were recruited between 2005 and 2009. Inclusion criteria were women and men between the ages of 35 and 70 who were willing to participate, intended to continue living in the same household for the next four years, and signed the informed consent. To obtain adequate geographic and social representation, participants were selected from Colombian urban and rural communities in 11 departments comprising 51.2% of the country’s population. For the present analysis, 7,552 participants were included. Local research ethics committees approved the study.

### 2.2 Data collection

Data collection was performed door-to-door by trained fieldworkers of the study team. A family census form recorded data on demographics, smoking, education, and medical history. Physical examination and anthropometric, blood pressure, and grip strength measurements were performed using the methods established by the PURE study protocol [18]. A 10 ml fasting blood sample was obtained to determine biochemical parameters, including lipid profile and glycemia.

### 2.3 Covariates

Behavioral risk factors were smoking (current or former versus never), alcohol consumption (current or former versus never), physical activity (measured using the long-form International Physical Activity Questionnaire), and diet calculated using the AHEI (alternate healthy eating index) score [19]. Low physical activity was defined as having done less than 600 metabolic equivalents × min per week. Psychosocial risk factors were education, categorized by education level (primary or low level versus secondary or university). Hypertension was defined as a systolic blood pressure (SBP) of at least 140 mmHg, diastolic blood pressure of at least 90 mmHg, self-reporting of hypertension history, or blood pressure medication use. Diabetes was defined as a baseline fasting plasma glucose concentration of 126 mg/dL or higher or a self-reported history of diabetes. Low grip strength was defined as being in the lowest tertile of grip strength by sex. We also calculated the grip strength adjusted by weight, determining low grip adjusted by weight as being in the lowest tertile calculated by sex. High body mass index (BMI) was defined as being at least 25 kg/m2. Elevated non-HDL cholesterol was defined as a fasting blood concentration of at least 171 mg/dL, which represented the upper third of the distribution. High triglycerides were defined as a fasting concentration of at least 150 mg/dL. The triglycerides glucose (TyG) index was determined using the formula Ln [fasting triglycerides (mg/dL) × fasting plasma glucose (mg (dL)/2].

### 2.4 Outcomes

The primary outcome was defined as the time to event of the composite of MACE, including death, cardiovascular death, myocardial infarction, stroke, or heart failure, whichever occurred first. In addition, the incidence of diabetes was assessed. Every three years, information on clinical events was obtained through face-to-face or telephone visits from the participants or relatives of the deceased participants. Events were collected using standardized case-report forms and based on autopsy information, medical records and hospital or physician reports and were adjudicated using standardized definitions (**Appendix A1, Supplementary material**). The outcome variables were taken with a censoring cut-off of February 28, 2021.

### 2.5 Statistical analysis

Continuous variables are presented as means and standard deviations. Categorical variables are presented as absolute frequencies and percentages. The differences in baseline data were tested by sex. A t-test was used to examine the differences between continuous variables and Pearson’s chi-square test between categorical variables. Kaplan-Meier curves were used to assess the cumulative incidence of outcomes differentiated by sex, and curves were compared employing the log-rank test. We calculated incidence rates for the MACE composite for each outcome and diabetes. The association between each risk factor of the baseline population and the MACE composite outcome was analyzed using the Cox proportional hazards model with random effects, with 95% confidence intervals (CI), including interaction terms between each variable and sex. The models were adjusted by age and location. The proportional hazards assumption was checked by visual inspection of log-to-log plots and by scaled Schoenfeld residuals (**Figures S1–S6, Supplementary material**). The random effects were included to account for the clustering of participants in the Colombian region’s denominated departments. Statistical analysis was performed using the survival library version 3.3-1 and the coxme library version 2.2-18.1 from the R software version 4.2.1.

## 3. Results

### 3.1 Population characteristics and risk factors at baseline and follow-up

Table 1 shows baseline characteristics. Of 7,552 participants, 64% were women, the mean baseline age was 50 (SD 9.5), and 53% lived in rural areas. Among traditional risk factors, women had a higher prevalence of hypertension (38.6% vs. 36.1%, p = 0.037) but lower levels of SBP (127 mmHg vs. 131 mmHg, p < 0.001). The prevalence of diabetes (5.9% vs. 5.1%, p = 0.162) was similar. In addition, women had higher BMI (26.8 kg/m2 vs. 25.3 kg/m2, p < 0.001). Likewise, in women, there were higher levels of total cholesterol, LDL-c, and HDL-c and lower triglyceride levels. Regarding lifestyle habits, women had lower levels of physical activity, active smoking and alcohol consumption. Women had a higher educational level (secondary education 20.8% vs. 17.6%, p = 0.003) and less frequently lived in rural areas (49.1% vs. 61.5%, p < 0.001). At 12 years (SD 2.3) follow-up, the prevalence of risk factors changed; however, baseline differences between both sexes remained (**Table S1, Supplementary material**). Alcohol consumption and smoking decreased but remained higher in men. The entire population increased the BMI, remaining higher in women. Hypertension prevalence increased and continued to be higher in women, but men had higher SBP levels. The prevalence of diabetes doubled, being higher in women (12.7% vs. 10.2%, p = 0.002). However, glucose levels increased similarly in both. Triglyceride levels remained stable in women but exhibited a decrease in men, resulting in the statistical difference between genders disappearing (p = 0.280).

**Table 1:** Population characteristics and risk factors at baseline.


BASELINE	WOMEN	MEN	TOTAL	*P* – VALUE

**Total – no. (%)**	4845 (64.2)	2707 (35.8)	7552 (100)	

**Age in years (SD)**	50.5 (9.5)	51.1 (9.9)	50.7 (9.7)	**0.021**

**Location – no. (%)**				

**Rural**	2378 (49.1)	1665 (61.5)	4043 (53.5)	**<0.001**

**Urban**	2467 (50.9)	1042 (38.5)	3509 (46.5)	

**Education – no. (%)**				

**Primary School or Unknown**	3142 (65.0)	1846 (68.2)	4988 (66.2)	**0.003**

**High School**	1004 (20.8)	476 (17.6)	1480 (19.6)	

**University**	688 (14.2)	384 (14.2)	1072 (14.2)	

**Civil status – no. (%)**				

**Never married**	663 (13.7)	315 (11.7)	978 (13.0)	**<0.001**

**Married – Common Law**	3187 (66.1)	2181 (80.8)	5368 (71.4)	

**Widowed / Divorced**	974 (20.2)	202 (7.5)	1176 (15.6)	

Alcohol consumption* – no. (%)	784 (16.2)	1344 (49.6)	2128 (28.2)	**<0.001**

**Tobacco use† – no. (%)**	446 (9.2)	599 (22.2)	1045 (13.9)	**<0.001**

**Low physical activity‡ – no. (%)**	803 (17.5)	442 (18.2)	1245 (17.7)	**<0.001**

**AHEI score (mean; SD)**	37.33 (7.10)	36.92 (7.31)	37.18 (7.18)	**0.021**

**Waist circumference (cm; SD)**	85.2 (11.6)	88.3 (11.1)	86.3 (11.5)	**<0.001**

**Body mass index (kg/m2; SD)**	26.8 (4.9)	25.2 (4.1)	26.2 (4.6)	**<0.001**

**Waist–to-Hip Ratio (mean; SD)**	0.86 (0.08)	0.93 (0.09)	0.88 (0.09)	**<0.001**

**Relative grip strength§ (mean; SD)**	0.36 (0.13)	0.50 (0.16)	0.41 (0.16)	**<0.001**

**Systolic blood pressure (mmHg; SD)**	127.3 (22.3)	131.0 (20.9)	128.6 (21.9)	**<0.001**

**Diastolic blood pressure (mmHg; SD)**	80.4 (12.6)	81.2 (12.9)	80.7 (12.7)	**0.018**

**Glucose (mg/dL; SD)**	86.8 (30.5)	86.7 (25.9)	86.7 (28.7)	**0.901**

**Total cholesterol (mg/dL; SD)**	204 (46.1)	196.3 (45.9)	200.8 (46.1)	**<0.001**

**HDL-c (mg/dL; SD)**	44.0 (10.2)	40.9 (10.0)	42.7 (10.2)	**<0.001**

**Non-HDL-c (mg/dL; SD)**	160.1 (42.9)	155.2 (42.1)	158.0 (42.6)	**<0.001**

**Triglycerides (mg/dL; SD)**	176.4 (103.7)	195.0 (120.9)	184.0 (111.5)	**<0.001**

**Hypertension – no. (%)**	1869 (38.6)	977 (36.1)	2846 (37.7)	**0.037**

**Diabetes – no. (%)**	286 (5.9)	138 (5.1)	424 (5.6)	**0.162**

**History of CVD – no. (%)**	232 (4.8)	122 (4.5)	354 (4.7)	**0.617**


AHEI: alternate healthy eating index; CVD: Cardiovascular disease; HDL-c: high-density lipoprotein; LDL-c: low density lipoprotein; SD: standard deviation. *Alcohol consumption was defined has alcohol consumption at least once a year. †Tobacco use was defined as current or previous tobacco use. ‡Low physical activity was defined as less than 600 metabolic equivalent × minutes per week. §Relative grip strength was calculated by dividing the absolute handgrip strength by body mass index.

### 3.2 Cardiovascular outcomes

**Figure 1:**
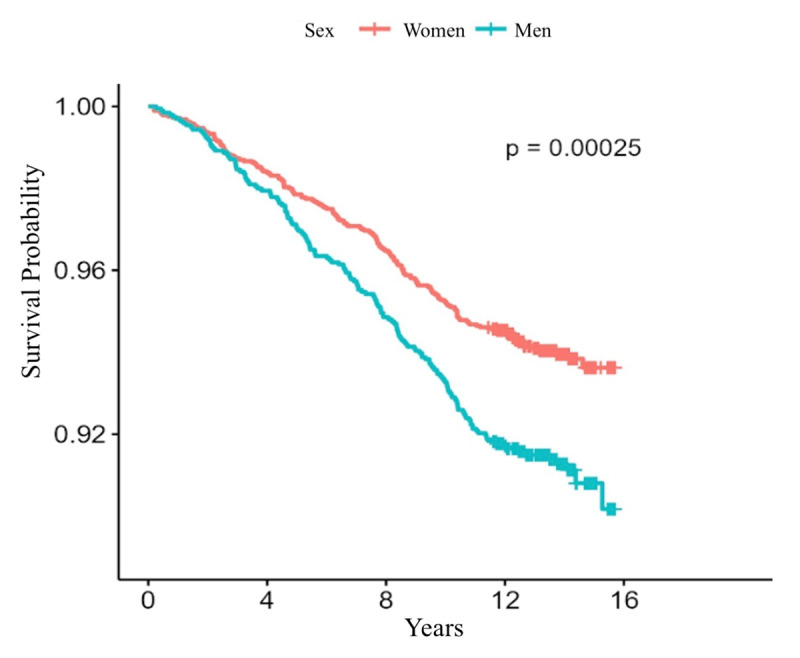
Kaplan-Meier curves of fatal cardiovascular events.

**Figure 2:**
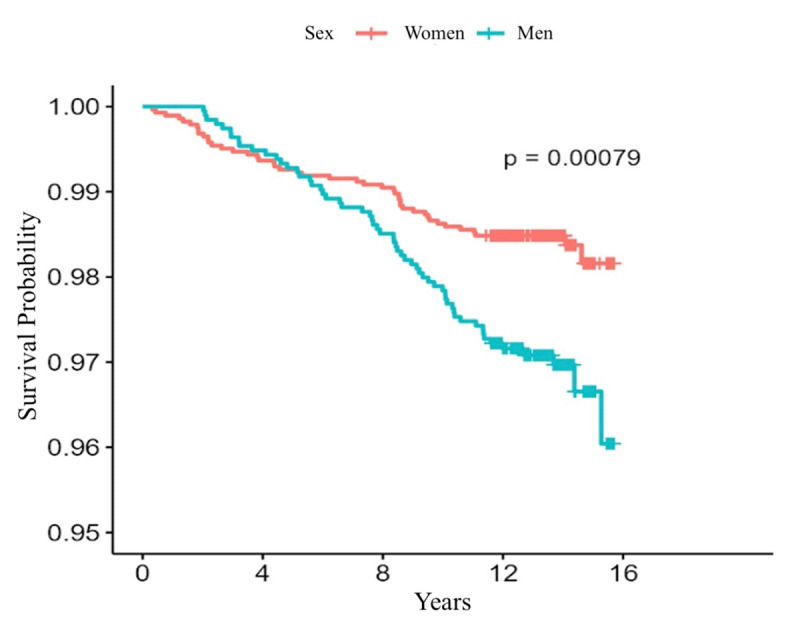
Kaplan-Meier curves for all-cause mortality.

Table 2 shows the incidence rates of cardiovascular outcomes during follow-up. There were 501 deaths [5.1 (4.7 to 5.6) cases per 1000 person-years], 352 cases of the MACE composite [3.6 (3.2 to 4) cases per 1000 person-years], and 340 cases of incident diabetes [3.4 (3.1 to 3.8) cases per 1000 person-years]. The incidence rate of MACE composite was higher in men [4.2 (3.6 to 4.9) vs. 3.2 (2.8 to 3.7) cases per 1000 person-years] as well as individual components of cardiovascular death [1.8 (1.4 to 2.2) vs. 0.9 (0.7 to 1.2) cases per 1000 person-years], and acute myocardial infarction [2.7 (2.2 to 3.3) vs. 1.7 (1.4 to 2.0) cases per 1000 person-years] with evident divergence in Kaplan-Meier curves reaching statistical significance (p < 0.001) (Figures 1 and 2). Meanwhile, a lower rate of diabetes was reported in men [2.7 (2.2 to 3.3) vs. 3.8 (3.4 to 4.3) cases per 1000 person-years]. Heart failure and stroke rates were similar.

**Table 2:** Incidence rates of composite MACE outcome and individual outcomes during the period 2009–2021.


	INCIDENCE RATE CASES PER 1000 PERSON-YEARS (95% CI)					

	EVENTS (N)	WOMEN	EVENTS (N)	MEN	EVENTS (N)	TOTAL

Composite MACE*	204	3.2 (2.8 to 3.7)	148	4.2 (3.6 to 4.9)	352	3.6 (3.2 to 4.0)

**Death**	276	4.4 (3.9 to 4.9)	225	6.5 (5.7 to 7.4)	501	5.1 (4.7 to 5.6)

**Cardiovascular death**	60	0.9 (0.7 to 1.2)	63	1.8 (1.4 to 2.2)	123	1.2 (1.0 to 1.5)

**Acute myocardial infarction**	107	1.7 (1.4 to 2.0)	95	2.7 (2.2 to 3.3)	202	2.0 (1.8 to 2.3)

**Heart failure**	53	0.8 (0.6 to 1.1)	27	0.8 (0.5 to 1.1)	80	0.8 (0.6 to 1.0)

**Stroke**	58	0.9 (0.7 to 1.2)	40	1.1 (0.8 to 1.5)	98	1.0 (0.8 to 1.2)

**Diabetes**	242	3.8 (3.4 to 4.3)	98	2.7 (2.2 to 3.3)	340	3.4 (3.1 to 3.8)


MACE: Major adverse cardiovascular events. *Composite of MACE defined as cardiovascular death, myocardial infarction, stroke, or heart failure, whichever occurred first.

### 3.3 Association between risk factors and cardiovascular outcomes

**Figure 3:**
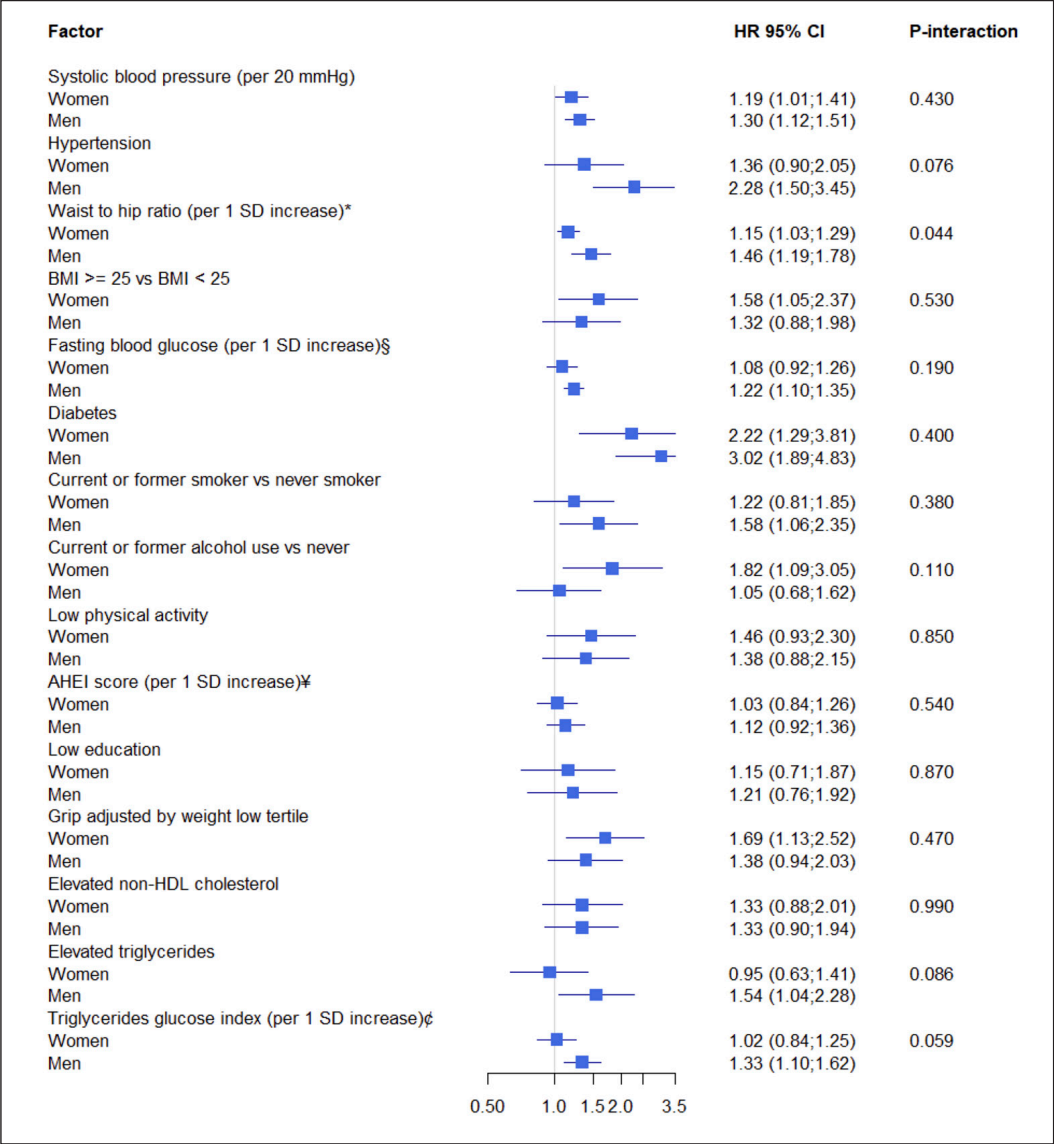
Associations between cardiovascular risk factors and MACE in women and men. *Note*: HR = hazard ratio. *1 SD increase in waist-to-hip ratio is 0.09. § 1 SD increase in fasting blood glucose is (27.94 mg/dL). ¥ 1 SD increase in AHEI score is 7.32. ¢ 1 SD increase in triglycerides glucose index is 0.57.

The association between the principal cardiovascular risk factors and the risk of MACE composite is shown in **Figure S7 (Supplementary material)**. Diabetes had the greatest association (HR = 2.63 95%CI: 1.85; 3.76) followed by hypertension (HR = 1.75 95%CI: 1.30; 2.35), low relative grip strength (HR = 1.53 95%CI: 1.15; 2.02), smoking (HR = 1.47 95%CI: 1.11; 1.93), low physical activity (HR = 1.42 95%CI: 1.03; 1.96). Increased AHEI diet score and low education were not associated with increased risk of MACE. When evaluating risk factors by sex, an increased waist-to-hip ratio was more strongly associated with MACE in men than in women. Meanwhile, in the rest of the risk factors, the HRs were similar between women and men (Figure 3). When performing a sensitivity analysis excluding participants with a history of CVD, the associations between risk factors and MACE maintained a similar trend, even when evaluating the differences between women and men (**Figure S8, S9, Supplementary material**).

## 4. Discussion

The present study demonstrates that after an average 12-year follow-up, men exhibited higher incidences of overall and cardiovascular death, the composite MACE outcome, and myocardial infarction despite having a lower baseline risk factor burden. In addition, when we evaluated the associations between CVD risk factors and MACE differentiated by sex, the HRs were similar. Although women presented a higher prevalence of diabetes, this did not additionally contribute to a higher incidence of MACE. Our results lead us to suggest reconsidering the need to carrying out differentiated cardiovascular risk prevention and management campaigns, at least in Colombia.

In the Framingham cohort, with about 5,000 participants, men had a higher prevalence of risk factors, and the incidence of sudden death (mainly due to coronary heart disease) was substantially higher [20]. In the Health Survey for England carried out between 2012–2017, a higher prevalence of smoking, hypertension, overweight, and dyslipidemia was shown in men [21]. Reports in HICs have demonstrated that the prevalence of risk factors has increased in both sexes, as well as the incidence of CVD in people under 55 years. For example, in the Atherosclerosis Risk in Communities (ARIC) study, the rate of myocardial infarction in younger patients was more pronounced in women, yet all-cause mortality was comparable between the sexes (HR = 1.10; 95%CI: 0.83; 1.45) [22]. It has been related to the fact that women experience greater delays in the diagnostic and therapeutic process of cardiovascular pathologies (e.g., myocardial revascularization) that could potentially be explained by differences in sex (biological and anatomical factors) and or gender (sociocultural factors) [8,23].

In LMICs, the PURE study [17] showed that the burden of risk factors and CVD was lower in women, regardless of the economic status of the countries, geographic regions, or history of CVD. When adjusting for the risk factor burden, the rate of MACE and death continued to be lower in women. Additionally, the INTERHEART case-control study that evaluated 27,098 participants, including Latin American countries (Argentina, Brazil, Colombia, Chile, Guatemala, and Mexico), demonstrated that in women, age of presentation of a first myocardial infarction was nine years later compared to men (65 vs. 56 years; p < 0.0001), mainly explained by the greater burden of risk factors at younger ages in men, and it was suggested that cardiovascular prevention measures should start earlier in men [24].

In the present analysis, women had a higher prevalence of hypertension, higher BMI, higher cholesterol levels, and lower levels of physical activity, but men had a higher prevalence of smoking and alcohol consumption and lower educational levels. A higher BMI could explain the contribution of these metabolic risk factors in women. This contribution of risk factors is quite like that shown in an observational study of 5 sub-Saharan African countries that included 15,356 and demonstrated that women had a poorer cardiovascular health status compared to men, mainly derived from a higher prevalence of hypertension (21.6% vs. 13.8%) and overweight/obesity (48.3% vs. 27.5%) [25]. In our study, although differences in the prevalence of risk factors were maintained at follow-up, no specific association was found that could explain the higher incidence in outcomes in men. The higher prevalence of hypertension in women persisted, even though, on average, their SBP levels were lower compared to men.

A higher incidence of diabetes was found in women despite the similar average glycemia values. These results suggested that there is an underdiagnosis or less self-reporting of diseases, less control of risk factors in men, and greater access to medical care in women. To support these suggestions, it is known that in studies where diabetes is self-reported, its association with CVD is greater. The UK Biobank study (n = 471,998) found that self-reported diabetes in women was associated with a higher incidence of myocardial infarction than in men despite similar HbA1c levels. Moreover, an increase of 18% in myocardial infarction was associated with a 1% increase in HbA1c in both sexes equally [6]. The PURE Global study showed that, in primary prevention, women are more likely to use preventive medications, have higher levels of hypertension control, and have less smoking, leading to a lower risk of CVD [17,26]. Furthermore, a meta-analysis from the Cholesterol Treatment Trialists’ (CTT) Collaboration evaluating 22 clinical trials with 175,000 subjects showed that statin therapy has similar efficacy for CVD prevention in men and women with equivalent cardiovascular risk [27]. These findings suggest the existence of factors other than biological characteristics *per se*. These factors are likely related to differences in sociocultural behaviors that could delay access to health services, prevention, diagnosis, and early treatment of CVD risk factors and the adherence to lifestyle modification and acceptance of pharmacological treatment.

It has been suggested that women are protected from MACE by their estrogen levels and that when these decrease, cardiovascular risk increases, mainly due to an increase in cholesterol levels. However, large clinical trials evaluating the effect of estrogens have not shown benefits, so the role of estrogens in protecting women remains controversial [9,10]. Furthermore, previous studies have demonstrated a correlation between the rise in lipid levels and aging [28]. However, it remains unclear whether menopause itself is associated with an elevated risk of CVD [29]. The educational level was significantly lower in men. Low education has been shown to affect the control of CVD risk factors driven by a predisposition to higher poverty index conditions, a lower possibility of access to health services, less possibility of employment, and, therefore, lower income. In the population of PURE-Colombia, we demonstrated that differences in hypertension rates between ethnic groups are mediated by educational level [30].

Our study has several strengths. Being a prospective cohort, it allows for the assessment of temporal relationships between an extensive collection of demographics, behavioral, and metabolic risk factors and their associations with MACE and death, as well as the establishment of potential differences between sexes. It is one the largest studies to date conducted in the Colombian population, with a substantial number of participants, enhancing the statistical power to detect differences and draw meaningful conclusions. The average follow-up period of 12 years is relatively long, providing valuable insights into the long-term outcomes of CVD risk factors in men and women. This study, however, does not escape limitations. The study population is limited to PURE-Colombia, which may not represent the diversity of sociocultural and demographic factors found in other regions, limiting the generalizability of the findings to other populations. However, as presented earlier, our results are in line with further research conducted in Latin American regions and LMICs, with differences found mainly when compared against HICs. Certain non-traditional and sociocultural cardiovascular risk factors might not have been collected in the data, limiting potential conclusions about their relationship with the sex differences and outcomes. Nevertheless, in the multivariate analysis conducted with plenty of demographic, lifestyle, anthropometric, and laboratory variables, no single risk factor was responsible for the differences found. Sixty-five percent of the population were women, affecting the sample’s representativeness. However, this information contributes to characterizing the presence of risk factors in women, usually underrepresented in clinical trials [31]. In addition, some risk factors were self-reported, which may introduce bias. Despite this, many variables and evaluations were derived from objective or adequately validated measurement instruments.

## 5. Conclusion

The incidence of MACE, total, and cardiovascular death was higher in men despite having a lower overall burden of risk factors at the baseline. In addition, different cardiovascular and psychosocial risk profiles were found between men and women; however, only the waist-to-hip ratio was found to increase the risk of the composite outcome when the analysis was differentiated by sex. Therefore, it suggests the existence of biological, psychosocial, or behavioral factors that could explain the difference in results and the apparent discrepancy with other international studies. Furthermore, the above makes us reconsider the need to carry out differentiated cardiovascular risk prevention and management campaigns, at least in Colombia. This holds important implications for future endeavors in research, clinical practice, and public policy, underscoring the necessity for in-depth research to elucidate the factors responsible for the elevated incidence of cardiovascular events in men despite their lower baseline risk factors.

## Data Accessibility Statement

Individual-level data will not be shared because PURE is an ongoing cohort study. Requests for aggregate data will be considered on a case-by-case basis on receipt of a reasonable request.

## Additional File

The additional file for this article can be found as follows:

10.5334/gh.1289.s1 Supplementary material. Appendix A1.
