# Supplementary material for: Sex Differences in Cardiovascular Disease Risk Factor Prevalence, Morbidity, and Mortality in Colombia: Findings from the Prospective Urban Rural Epidemiology (PURE) Study
Source: Glob Heart. 2024 Jan 24;19(1):10. doi: 10.5334/gh.1289 (PMC10809858; doi:10.5334/gh.1289)
Supplement: Supplementary material. — Appendix A1. [file gh-19-1-1289-s1.pdf]

Supplementary material

“**Sex differences in cardiovascular disease risk factor prevalence, morbidity, and mortality in Colombia: findings from the Prospective Urban Rural Epidemiology (PURE) study**”

Appendix A1: Standardized Event Definitions in PURE. from Teo et al. Am Heart J. 2009 Jul;158(1):1-7

Prospective Follow-up for Cardiovascular Events and Mortality: History of disease was collected at baseline from every participant with standardized questionnaires regarding history of a) hypertension, b) diabetes c) stroke d) angina/myocardial infarction/coronary artery disease e) heart failure f) other heart disease.

Information on specific events (death, myocardial infarction, stroke, heart failure, cancer, hospitalizations, new diabetes, injury, tuberculosis, human immunodeficiency viral infections, malaria, pneumonia, asthma, chronic obstructive pulmonary disease) were obtained from participants or their family members (events were reported by the participants if alive or by a relative if the individual had died). This information was adjudicated centrally in each country by trained physicians using standardized definitions. Because the PURE study involves urban and rural areas from middle- and low- income countries, supporting documents to confirm cause of death and/or event varied in degrees of completion and availability. In most of middle- and low-income countries there was no central system of death or event registration. Therefore, information was obtained about prior medical illness and medically certified cause of death where available, and, second, best available information was captured from reliable sources in those instances where medical information was not available in order to be able to arrive at a probable diagnosis or cause of death. Event documentation was based on information from household interviews and medical records, death certificates and other sources. Verbal autopsies were also used to ascertain cause of death in addition to medical records which were reviewed by a health professional. This approach has been used in several studies conducted in middle- and low-income countries.

FATAL EVENTS

Cardiovascular Death – Definitions

01.00 DEATH DUE TO CARDIOVASCULAR EVENTS

01.10 Sudden unexpected Cardiovascular Death (SCVD)

Without evidence of other cause of death, death that occurred suddenly and unexpectedly (examples: witnessed collapse, persons resuscitated from cardiac arrest who later died) or

persons seen alive less than 12 hours prior to discovery of death (example persons found dead in his/her bed).

• SCVD is either definite, probable or possible according to the following characteristics:

<table>
<tr><th>PURE

Adjudication Code</th><th>Event Type</th><th>Acceptable ICD-10 codes</th></tr>
<tr><td>01.11: Definite</td><td>One of the following in persons with:
known cardiovascular disease, or
diabetes with an additional risk factor such as hypertension, smoking, dyslipidemia, micro albuminuria, serum creatinine 50% above upper limit of normal, or
3 of the above risk factors, or
2 of the above risk factors in men aged 60 and more and women aged 65 and more</td><td rowspan="3">No ICD-10 Code</td></tr>
<tr><td>01.12: Probable</td><td>One of the following in persons with:
diabetes, or
2 of the above risk factors in men aged less than 60 and in women less than 65, or
one of the above risk factor in men aged 60 and more and in women aged 65 and more, or
typical of chest pain or sudden severe dyspnea of less than 20- minute duration preceding the event</td></tr>
<tr><td>01.13: Possible</td><td>In persons without risk factor</td></tr>
<tr><td colspan="3">For SCVD, the patient was well or had a stable CVD (example stable angina) when last seen alive. The event of a sudden death occurring during the hospitalization of MI is considered a fatal MI and not sudden death.</td></tr>
</table>

01.30 Fatal Myocardial Infarction (MI)

Symptoms of Myocardial Infarction:

Typical symptoms or suggestive symptoms of MI according to physician are characterized by severe anterior chest pain as tightness, crushing, burning, lasting at least 20 minutes, occurring at rest, or on exertion, that may radiate to the arms or neck or jaw and may be associated with dyspnea, diaphoresis and nausea. However, death associated with nausea and vomiting with or without chest pain not due to another cause may be considered as possible MI if ECG and cardiac markers are not done. These symptoms may have occurred the last month before death.

Fatal myocardial infarction is either definite, probable or possible according to the following characteristics:

<table>
<tr><th>PURE

Adjudication Code</th><th>Event Type</th><th>Acceptable

ICD-10 codes</th></tr>
<tr><td>01.31: Definite</td><td>1. Autopsy demonstrating fresh myocardial infarction and/or recent coronary occlusion, or

2. ECG showing new and definite sign of MI (Minnesota code 1- 1-1) or

3. Symptoms typical or atypical or inadequately described but attributed to cardiac origin lasting at least 20 minutes and by troponin or cardiac enzymes (CKMB, CK, SGOT, SLDH) above center laboratory ULN

4. ECG with new ischemic changes (new ST elevation/depression or T wave inversion ≥ 2 mm) and by troponin or cardiac enzymes (CKMB, CK, SGOT, SLDH) above center laboratory ULN</td><td rowspan="2">I21- I22</td></tr>
<tr><td>01.32: Probable</td><td>1. ECG with sign of probable MI (Minnesota code 1-2-1), or</td></tr>
</table>

	2. Typical symptoms lasting at least 20 minutes considered of cardiac origin, with only new ST-T changes (new ST elevation/depression or T wave inversion ≥ 1 but < 2mm) without documented increased cardiac markers or enzyme as in PURE definition 1.31 (above), or<br><br>3. Increased cardiac enzymes as in PURE definition 1.31 (above) showing a typical pattern of MI as above without symptoms or significant ECG changes<br>.	
01.33: Possible	1. ECG with sign of possible MI (Minnesota code 1-3-1) or<br><br>2. Typical symptoms or symptoms suggestive of MI according to the physician lasting at least 20 minutes without documented ECG or cardiac marker.	

The Minnesota codes for MI is taken from Rose and Blackburn and published in their book "Evaluation Methods of Cardiovascular Disease WHO 1969".

- Definite MI is Q/R ratio ≥1/3 and Q duration ≥ 0.03 second in one of the following leads: I, II, V2, 3, 4, 5, 6. (code 1-1-1)
- Probable MI is Q/R ratio ≥1/3 and Q duration between 0.02 and 0.03 second in one of the following leads: I, II, V2, 3, 4, 5, 6. (code 1-2-1)
- Possible MI is Q/R ratio between 1/5 and 1/3 and Q duration between 0.02 and 0.03 second in one of the following leads: I, II, V2, 3, 4, 5, 6. (code 1-3-1)

<u>01.40 Fatal Stroke</u>
Fatal stroke is either definite or possible according to the following characteristics:

PURE<br><br>Adjudication Code	Event Type	Acceptable ICD-10 codes

01.41: Definite	Stroke death is defined as death within 30 days from an acute focal neurological deficit diagnosed by a physician and thought to be of<br><br>vascular origin (without other cause such as brain tumor) with signs and symptoms lasting >= 24 hrs.<br><br>Stroke death is also considered if death occurred within 24 hrs. of onset of persisting signs and symptoms, or if there is evidence of a recent stroke on autopsy.<br><br>N.B.<br><br>• In a subject with a stroke <= 30 days: If death occurred with a pneumonia due to possible aspiration, death will be considered to be due to stroke.<br>• In a subject with a stroke > 30 days: If death occurred with a pneumonia due to possible aspiration, the adjudicator will make a decision according to his/her clinical judgment if death is related to stroke or not.<br>• Subarachnoid hemorrhage death manifested by sudden onset headache with/without focal signs and imaging (CT or MRI) evidence of bleeding primarily in the subarachnoid space is considered a fatal stroke in absence of trauma or brain tumor or malformation<br>• Subdural hematoma death is not considered as a stroke death and may be related to previous trauma or other cause.	I60- I64, I69
01.43: Possible	Death in a participant with a history of sudden onset of focal neurological deficit of one or more limbs, loss of vision or slurred speech lasting about 24 hours.	

01.50 Fatal Congestive Heart Failure

Fatal congestive heart failure is either definite or possible according to the following characteristics:

PURE<br><br>Adjudication Code	Event Type	Acceptable ICD-10 codes
01.51: Definite	The diagnosis of congestive heart failure may be an autopsy finding in absence of other cause or requires signs (rales, increased jugular venous pressure or ankle edema) or symptoms (nocturnal paroxysmal dyspnea, dyspnea at rest or ankle edema) of congestive heart failure and one or both of the following:<br><br>• radiological signs of pulmonary congestion,<br>• treatment of heart failure with diuretics<br><br>If sudden death occurred in a patient with chronic severe heart failure, it should be adjudicated as fatal congestive heart failure.	I50
01.52: Probable	Progressive shortness of breath on lying down or at night, improving on sitting up AND any of the following signs or symptoms: swelling of feet, distension of abdomen, progressive cough in a person with known hypertension or a history of previous MI/angina or other heart disease	
	Progressive shortness of breath on lying down or at night, improving on sitting up AND any of the following signs or symptoms: swelling of feet, distension of abdomen, progressive cough	

01.60 Death Due to Other Cardiovascular Deaths (other causes [1.10 to 1.50 above] having been excluded)

PURE<br><br>Adjudication Code	Event Type	Acceptable ICD-10 codes
01.61	Arterial rupture of aneurysm	I71- I72
01.62	Pulmonary embolism<br><br>NOTE: Death associated with pulmonary embolism occurring within 2 weeks after a fracture such as hip, femur should attributed to death due to injury. Refer to Injury, Section 6.0	I26
01.63	Arrhythmic death (A-V block, sustained ventricular tachycardia in absence of other causes).	I44- I45, I47- I49
01.64	Death after invasive cardiovascular intervention: a perioperative death extending to 30 days after coronary or arterial surgical revascularization and to 7 days after a coronary or arterial percutaneous dilatation (angioplasty) with or without a stent or an invasive diagnostic procedure.	I97
01.65	Congenital heart disease	Q20-Q28
01.66	Heart valve disease (including rheumatic heart disease)	I01, I05- I09, I34- I37
01.67	Endocarditis	I33, I38
01.68	Myocarditis	I40
01.69	Tamponade (pericarditis)	I30,I31,I32

01.70	Other cardiovascular events (Excluding 1.61 to 1.69 above) Valid ICD-10 codes would include the following:<br><br>I11, I12, I13, I23, I24, I25, I27, I28, I42, I51, I52, I65-I68, I73, I74, I96, I98, I99 (Refer to ICD-10 Listing for associated definitions for each code)	Any valid 'I' (Cardiovascular) ICD-10 code that can be classified as underlying cause of death, not specified above

NON-FATAL EVENTS

Cardiovascular Events – Definitions

10.00 NON-FATAL CARDIOVASCULAR EVENTS

10.10 Non-Periprocedural Myocardial Infarction (MI)

MI is considered either definite, probable or possible according to the following characteristics:

PURE<br><br>Adjudication Code	Event Type	Acceptable ICD-10 codes
10.11: Definite	1. ECG showing new and definite sign of MI (Minnesota code 1- 1-1) or<br><br>2. Symptoms typical or atypical or inadequately described but attributed to cardiac origin lasting at least 20 minutes and by troponin or cardiac enzymes (CKMB, CK, SGOT, SLDH) above center laboratory ULN<br><br>3. ECG with new ischemic changes (new ST elevaton/depression or T wave inversion ≥ 2 mm) and by troponin or cardiac enzymes (CKMB, CK, SGOT, SLDH) above center laboratory ULN<br><br>Please note that increased markers may occur in trauma (CK, AST, myoglobin and CK MB to a lesser	

	degree); renal insufficiency, heart failure, pulmonary embolism (troponin), cardioversion (all)	I21-I22
10.12: Probable	1. ECG with new and probable sign of MI (Minnesota code 1-2- 1), or<br><br>2. Typical symptoms lasting at least 20 minutes considered of cardiac origin, with only new ST-T changes (new ST elevation/depression or T wave inversion ≥ 1 but < 2mm) without documented increased cardiac markers as in PURE definition 10.11 (above), or<br><br>3. Increased cardiac enzymes showing a typical pattern of MI as above without symptoms or significant ECG changes.	
10.13: Possible	1. ECG with new and possible sign of MI (Minnesota code 1-3- 1), or<br><br>2. Typical symptoms lasting 20 minutes and more considered to be of cardiac origin without documented ECG or cardiac marker.	

The Minnesota codes for MI is taken from Rose and Blackburn and published in their book "Evaluation Methods of Cardiovascular Disease WHO 1969".

- Definite MI is Q/R ratio ≥1/3 and Q duration ≥ 0.03 second in one of the following leads: I, II, V2, 3, 4, 5, 6. (code 1-1-1)
- Probable MI is Q/R ratio ≥1/3 and Q duration between 0.02 and 0.03 second in one of the following leads: I, II, V2, 3, 4, 5, 6. (code 1-2-1)
- Possible MI is Q/R ratio between 1/5 and 1/3 and Q duration between 0.02 and 0.03 second in one of the following leads: I, II, V2, 3, 4, 5, 6. (code 1-3-1)

10.30 Stroke/Transient Ischemic Attack (TIA)

PURE<br><br>Adjudication Code	Event Type	Acceptable ICD-10 codes
10.31: Definite	Stroke is defined as an acute focal neurological deficit diagnosed by a physician and thought to be of vascular origin (without other case such as brain tumor) with signs and symptoms lasting ≥ 24 hrs.<br><br>N.B.<br><br>• Subarachnoid hemorrhage manifested by sudden onset headache with/without focal signs and imaging (CT or MRI or lumbar puncture) showing evidence of bleeding primarily in the subarachnoid space is considered a stroke in absence of trauma or brain tumor or malformation<br><br>• Subdural hematoma is not considered as a stroke and may be related to previous trauma or other cause.	I60-I64, I69
10.33: Possible	Stroke is possible if there is a history of sudden onset of focal neurological deficit of one or more limbs, loss of vision or slurred speech lasting about 24 hours or more	
10.34: TIA	The diagnosis of TIA requires the presence of acute focal neurological deficit thought to be of vascular origin with signs and symptoms<br>lasting less than 24 hours.	G45

10.40 Congestive Heart Failure

PURE	Event Type	Acceptable ICD-10 codes

Adjudication Code		
10.41: Definite	The diagnosis of congestive heart failure requires signs (rales, increased jugular venous pressure or ankle edema) or symptoms (nocturnal paroxysmal dyspnea, dyspnea at rest or ankle edema) of congestive heart failure and one or both of the following:<br><br>• radiological signs of pulmonary congestion,<br>• Treatment of heart failure with diuretics.	
10.42: Probable	Progressive shortness of breath on lying down or at night, improving on sitting up AND any of the following signs or symptoms: swelling of feet, distension of abdomen, progressive cough in a person with known hypertension or a history of previous MI/angina or other heart disease	I50
10.43: Possible	Congestive heart failure is considered possible when there is progressive shortness of breath on lying down or at night, improving on sitting up AND any of the following signs or symptoms: swelling of feet, distension of abdomen, progressive cough	

Figure S1. Log-log plots of the variables included in the proportional hazard models for modeling the time to event of MACE, for men.

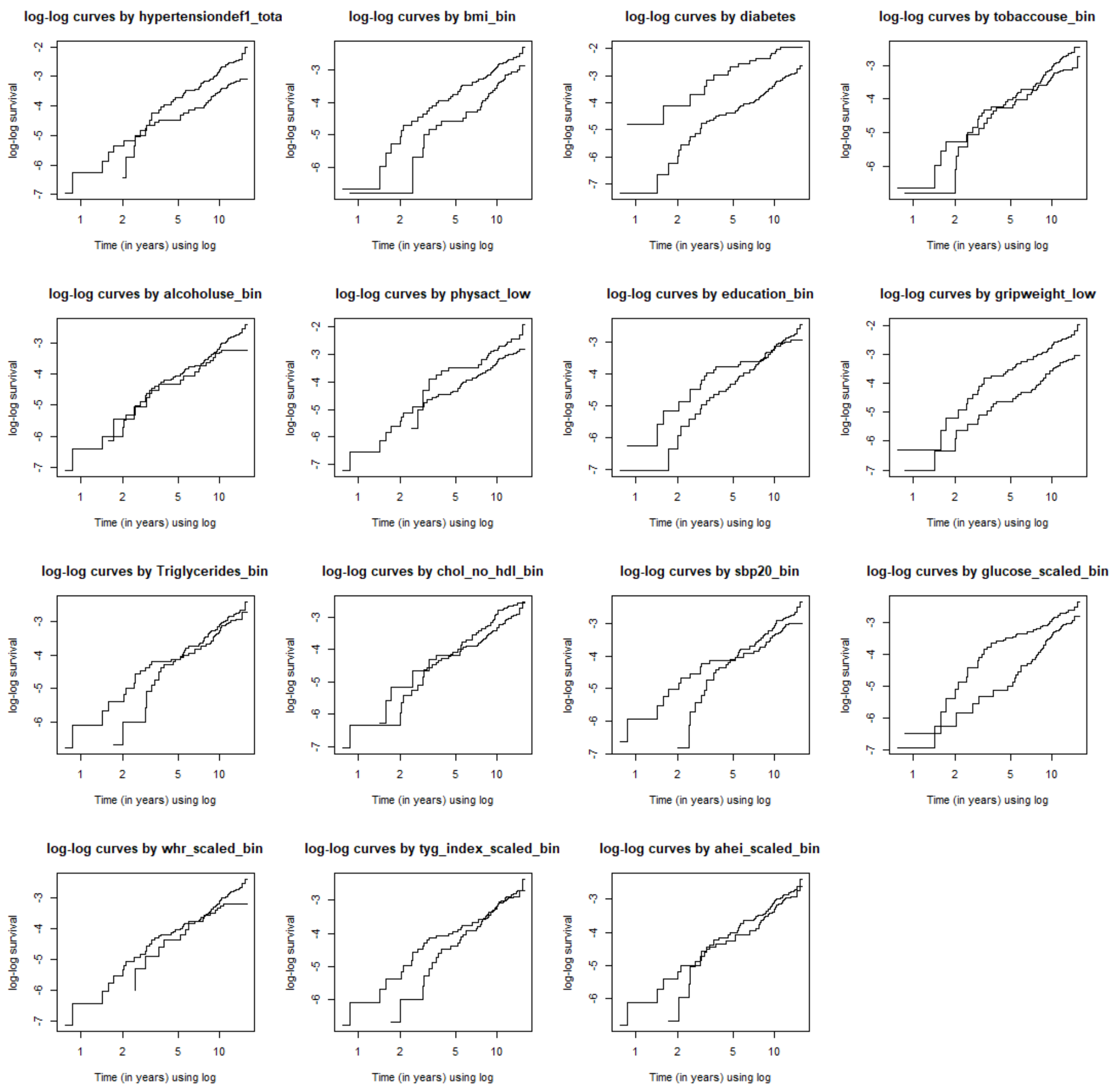

Figure S2. Log-log plots of the variables included in the proportional hazard models for modeling the time to event of MACE, for women.

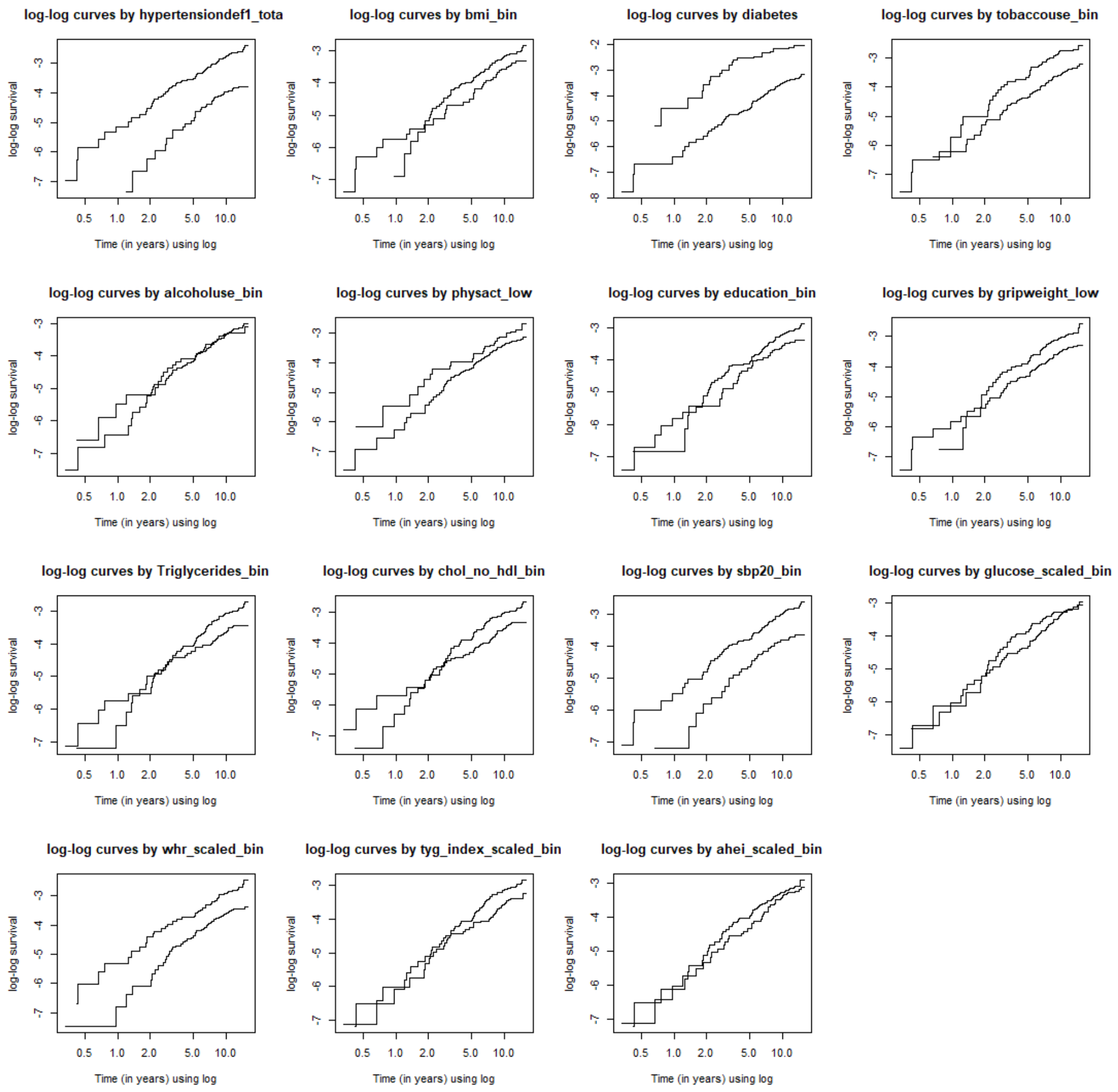

Figure S3. Scaled Schoenfeld residuals from the proportional hazards models for the time to event of major cardiovascular events for the variables (systolic blood pressure, hypertension, scaled waist to hip ratio scaled, BMI, glucose, diabetes, tobacco use, alcohol use and physical activity) in men.

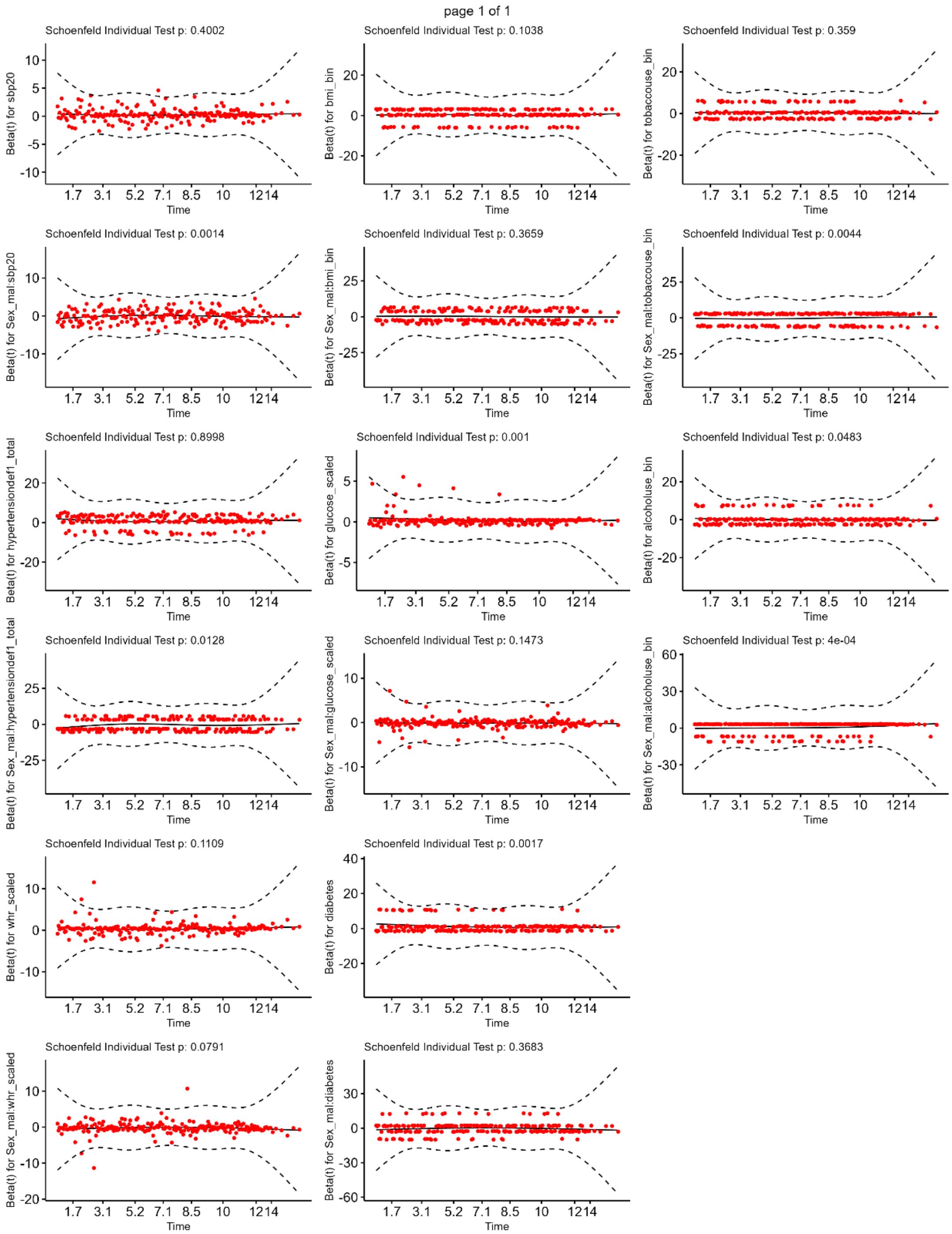

Figure S4. Scaled Schoenfeld residuals from the proportional hazards models for the time to event of major cardiovascular events for the variables (AHEI score, education, grip strength, grip adjusted by weight, cholesterol, triglycerides, LDL, and triglycerides glucose index) in men.

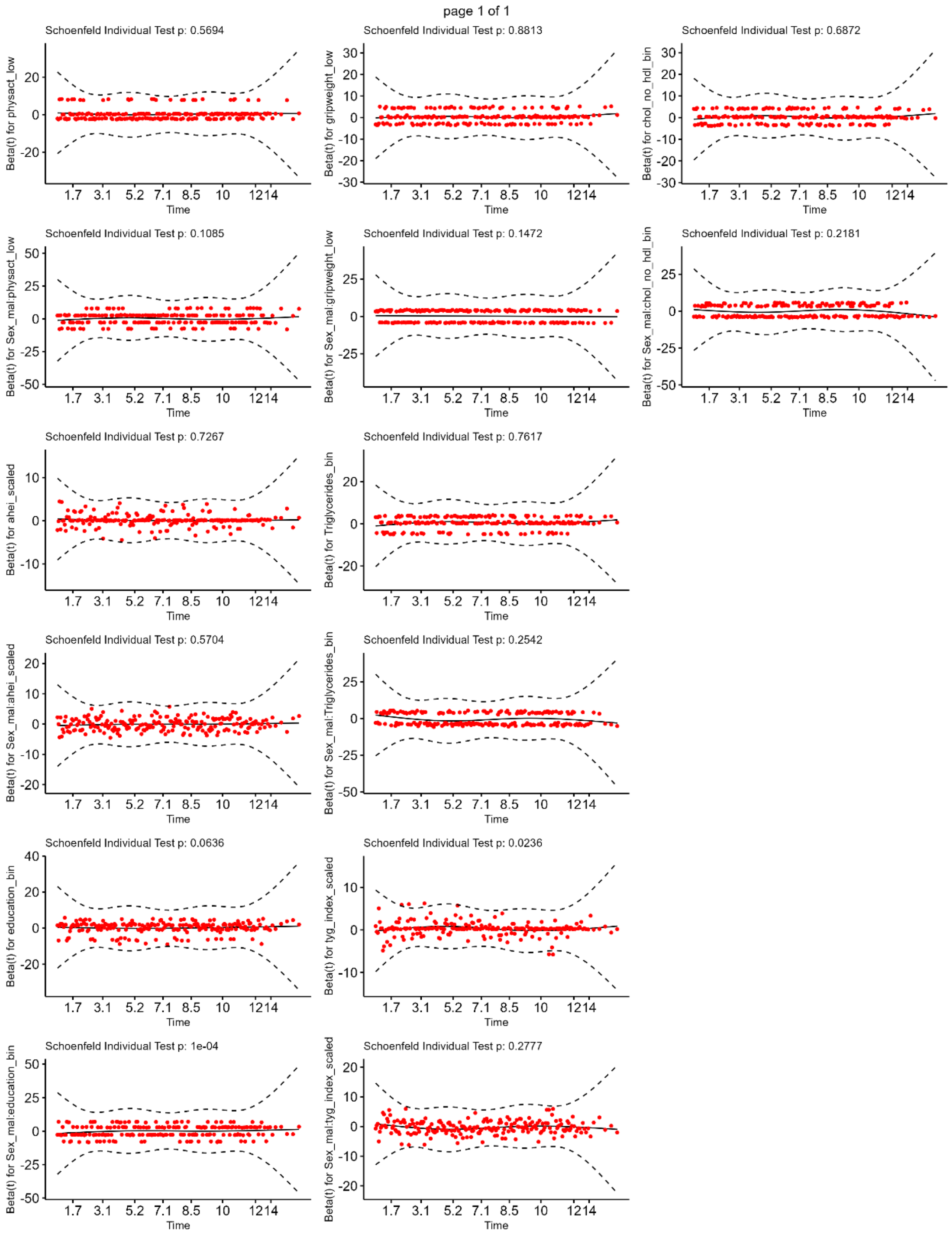

Figure S5. Scaled Schoenfeld residuals from the proportional hazards models for the time to event of major cardiovascular events for the variables (systolic blood pressure, hypertension, scaled waist to hip ratio scaled, BMI, glucose, diabetes, tobacco use, alcohol use and physical activity) in women.

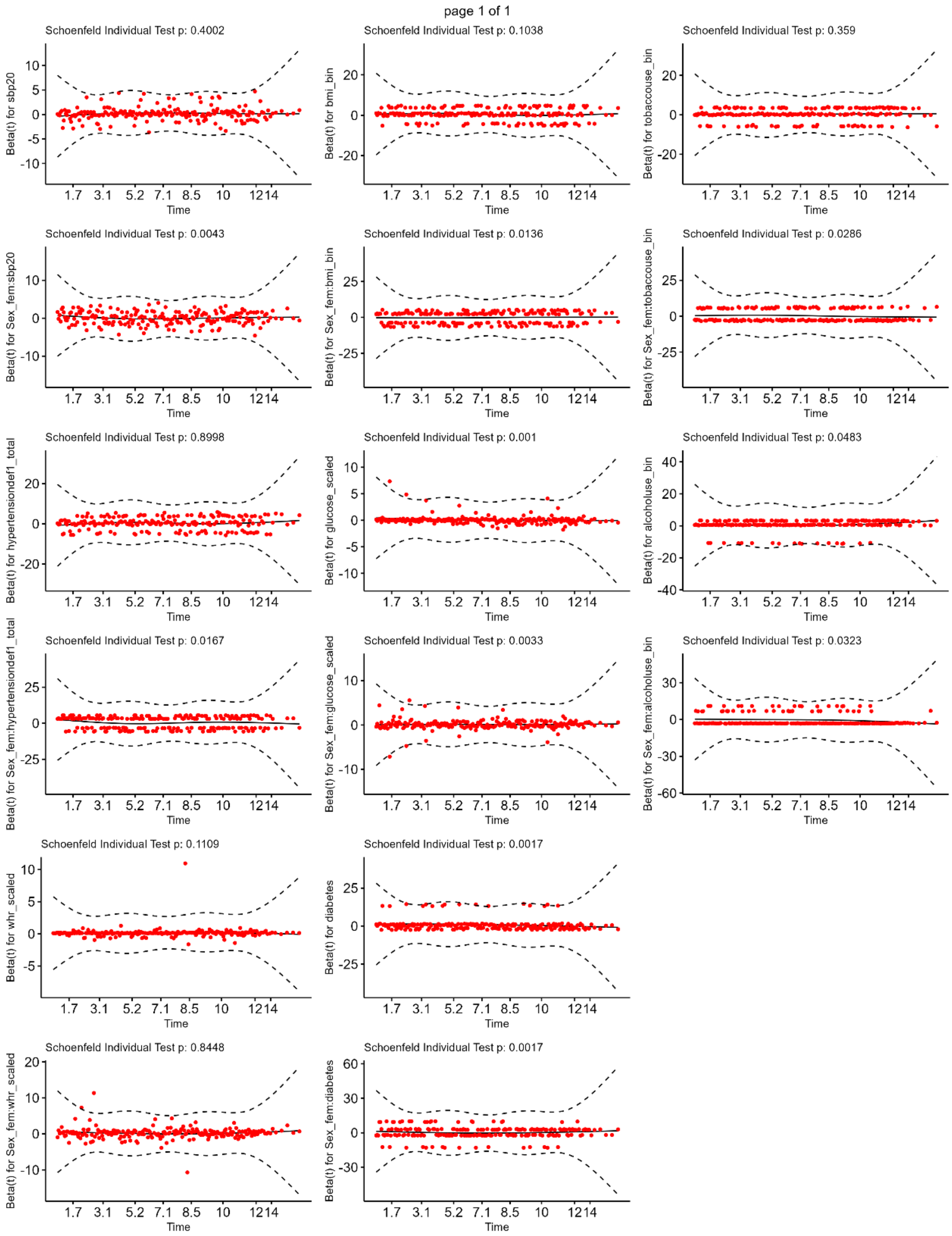

Figure S6. Scaled Schoenfeld residuals from the proportional hazards models for the time to event of major cardiovascular events for the variables (AHEI score, education, grip strength, grip adjusted by weight, cholesterol, triglycerides, LDL, and triglycerides glucose index) in women.

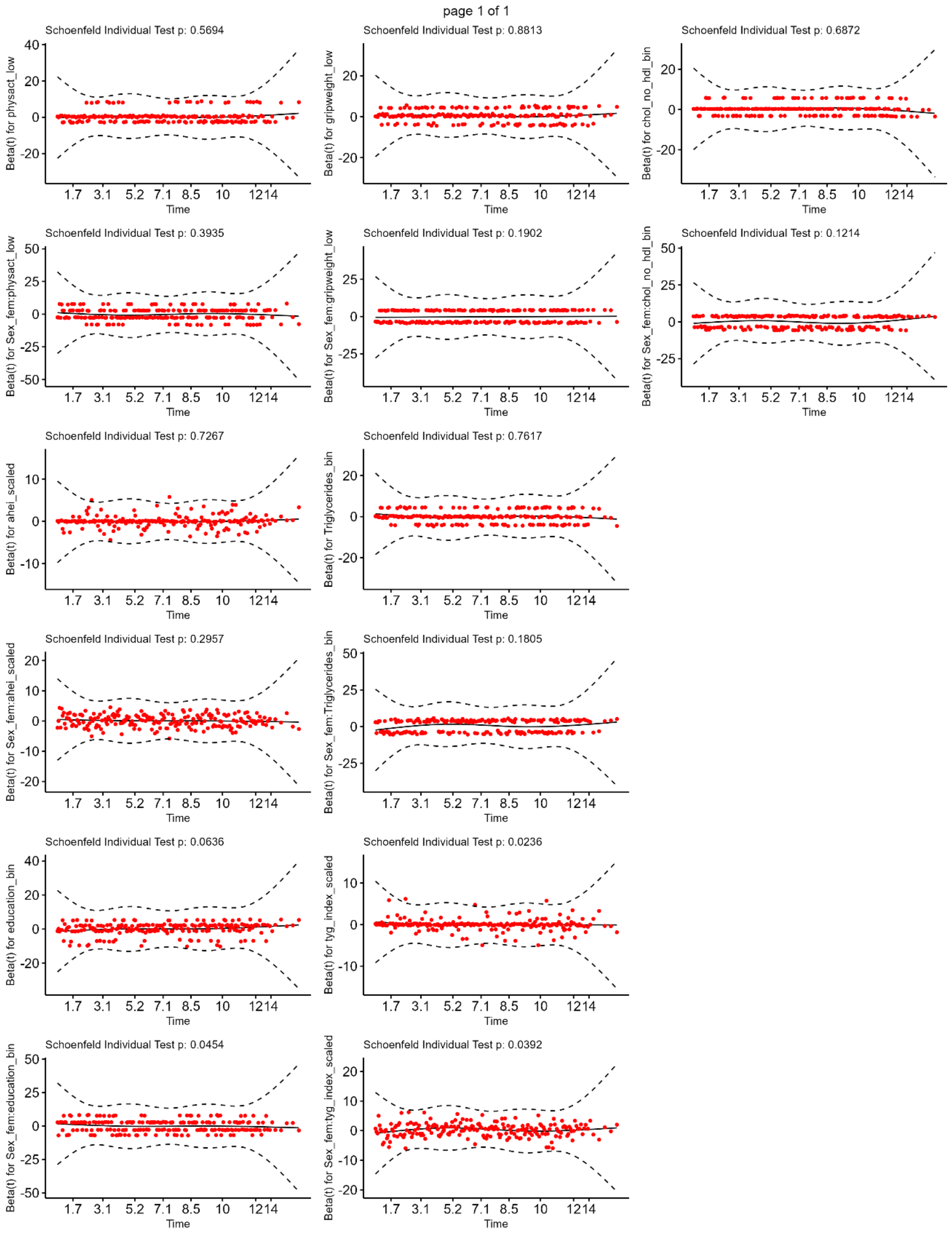

Figure S7. Associations between metabolic risk factors and cardiovascular disease in all population. Major cardiovascular disease is a composite of cardiovascular disease death, myocardial infarction, stroke, and heart failure. p values correspond to the term for the risk factor, adjusted by age and location. HR=hazard ratio. *1 SD increase in waist-to-hip ratio is 0.09. § 1 SD increase in fasting blood glucose is (27.94 mg/dL). ¥ 1 SD increase in AHEI score is 7.32. ¢ 1 SD increase in triglycerides glucose index is 0.57.

Factor	HR 95% CI	P-value
Systolic blood pressure (per 20 mmHg)	1.25 (1.12;1.40)	p<0.001
Hypertension	1.77 (1.32;2.38)	p<0.001
Waist to hip ratio (per 1 SD increase)*	1.20 (1.11;1.31)	p<0.001
BMI >= 25 vs BMI < 25	1.44 (1.08;1.93)	0.013
Fasting blood glucose (per 1 SD increase)§	1.16 (1.07;1.27)	0.001
Diabetes	2.62 (1.84;3.75)	p<0.001
Current or former smoker vs never smoker	1.40 (1.04;1.88)	0.026
Current or former alcohol use vs never	1.33 (0.97;1.82)	0.079
Low physical activity	1.42 (1.03;1.96)	0.034
AHEI score (per 1 SD increase)¥	1.07 (0.93;1.24)	0.340
Low education	1.18 (0.83;1.69)	0.360
Grip adjusted by weight low tertile	1.52 (1.15;2.01)	0.003
Elevated non-HDL cholesterol	1.33 (1.00;1.76)	0.048
Elevated triglycerides	1.22 (0.92;1.61)	0.160
Triglycerides glucose index (per 1 SD increase)¢	1.17 (1.02;1.34)	0.028

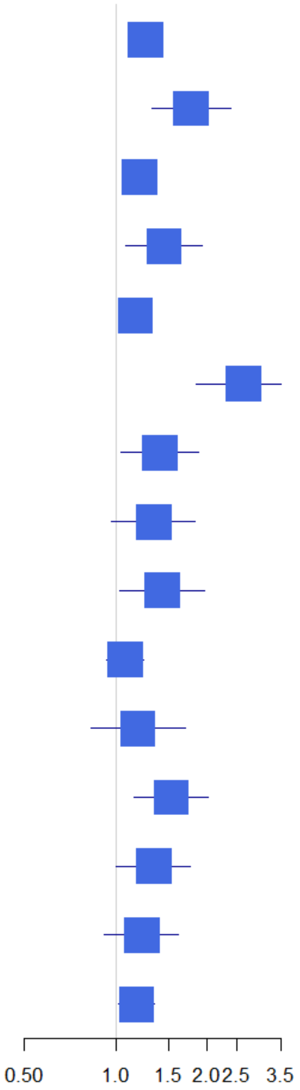

Figure S8. Sensitivity analysis. Associations between risk factors and cardiovascular disease in all participants excluding participants history of cardiovascular disease at baseline. Major cardiovascular disease is a composite of cardiovascular disease death, myocardial infarction, stroke, and heart failure. P-values correspond to the term for the risk factor, adjusted by age and location. HR=hazard ratio. *1 SD increase in waist-to-hip ratio is 0.09. § 1 SD increase in fasting blood glucose is (27.63 mg/dL). ¥ 1 SD increase in AHEI score is 7.35. ¢ 1 SD increase in triglycerides glucose index is 0.57.

Factor	HR 95% CI	P-value
Systolic blood pressure (per 20 mmHg)	1.26 (1.11;1.42)	p<0.001
Hypertension	1.64 (1.20;2.24)	0.002
Waist to hip ratio (per 1 SD increase)*	1.21 (1.08;1.35)	0.001
BMI >= 25 vs BMI < 25	1.46 (1.07;1.99)	0.018
Fasting blood glucose (per 1 SD increase)§	1.17 (1.06;1.28)	0.001
Diabetes	2.20 (1.45;3.32)	p<0.001
Current or former smoker vs never smoker	1.27 (0.92;1.74)	0.140
Current or former alcohol use vs never	1.35 (0.96;1.89)	0.084
Low physical activity	1.44 (1.02;2.03)	0.037
AHEI score (per 1 SD increase)¥	1.05 (0.90;1.23)	0.520
Low education	1.20 (0.81;1.77)	0.360
Grip adjusted by weight low tertile	1.33 (0.98;1.79)	0.063
Elevated non-HDL cholesterol	1.45 (1.07;1.96)	0.016
Elevated triglycerides	1.23 (0.91;1.65)	0.170
Triglycerides glucose index (per 1 SD increase)¢	1.16 (1.00;1.35)	0.052

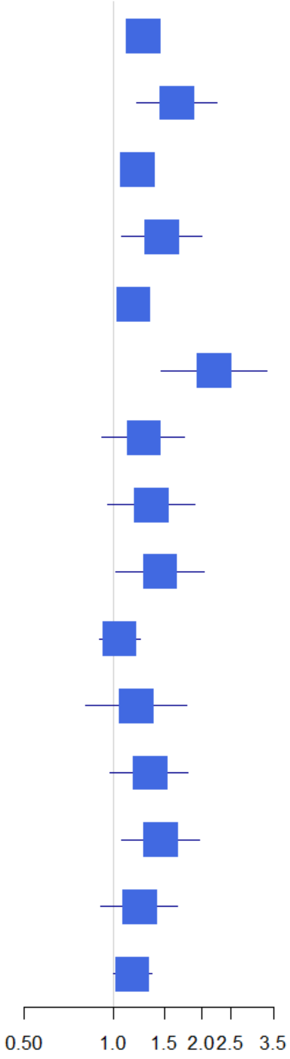

Figure S9: Sensitivity analysis. Associations between risk factors and cardiovascular disease by sex excluding participants history of cardiovascular disease at baseline. Major cardiovascular disease is a composite of cardiovascular disease death, myocardial infarction, stroke, and heart failure. P-values correspond to the term for the risk factor, adjusted by age and location. HR=hazard ratio. *1 SD increase in waist-to-hip ratio is 0.09. § 1 SD increase in fasting blood glucose is (27.63 mg/dL). ¥ 1 SD increase in AHEI score is 7.35. ¢ 1 SD increase in triglycerides glucose index is 0.57.

Factor	HR 95% CI	P-interaction
Systolic blood pressure (per 20 mmHg)		
Women	1.17 (0.97;1.41)	0.280
Men	1.33 (1.14;1.55)	
Hypertension		
Women	1.22 (0.79;1.88)	0.056
Men	2.20 (1.41;3.41)	
Waist to hip ratio (per 1 SD increase)*		
Women	1.11 (0.93;1.32)	0.037
Men	1.50 (1.20;1.88)	
BMI >= 25 vs BMI < 25		
Women	1.69 (1.09;2.60)	0.330
Men	1.25 (0.81;1.93)	
Fasting blood glucose (per 1 SD increase)§		
Women	1.12 (0.95;1.32)	0.490
Men	1.20 (1.07;1.34)	
Diabetes		
Women	1.97 (1.07;3.65)	0.640
Men	2.41 (1.38;4.19)	
Current or former smoker vs never smoker		
Women	1.08 (0.70;1.66)	0.290
Men	1.49 (0.97;2.31)	
Current or former alcohol use vs never		
Women	1.85 (1.07;3.20)	0.120
Men	1.05 (0.66;1.69)	
Low physical activity		
Women	1.47 (0.91;2.39)	0.900
Men	1.41 (0.88;2.26)	
AHEI score (per 1 SD increase)¥		
Women	1.03 (0.83;1.27)	0.740
Men	1.08 (0.87;1.33)	
Low education		
Women	1.11 (0.66;1.88)	0.680
Men	1.28 (0.77;2.13)	
Grip adjusted by weight low tertile		
Women	1.53 (1.00;2.34)	0.360
Men	1.16 (0.76;1.76)	
Elevated non-HDL cholesterol		
Women	1.42 (0.92;2.18)	0.890
Men	1.48 (0.98;2.23)	
Elevated triglycerides		
Women	0.92 (0.60;1.40)	0.060
Men	1.63 (1.07;2.48)	
Triglycerides glucose index (per 1 SD increase)¢		
Women	1.01 (0.82;1.25)	0.072
Men	1.33 (1.08;1.63)	

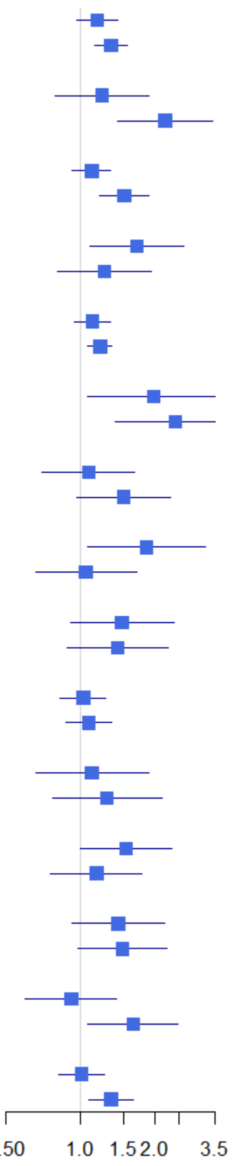

Table S1. Baseline and follow-up population characteristics.

Baseline	Women	Men	Total	*p*- value	Follow-up	Women	Men	Total	*p*- value
Age in years (SD)	50.5 (9.5)	51.1 (9.9)	50.7 (9.7)	**0.021**	Age in years (SD)	64.4 (9.6)	64.8 (9.8)	64.6 (9.7)	0.115
Alcohol consumption* (%)	784 (16.2)	1344 (49.6)	2128 (28.2)	**<0.001**	Alcohol consumption* (%)	304 (8.2)	650 (32.0)	954 (16.6)	**<0.001**
Tobacco use† (%)	446 (9.2)	599 (22.2)	1045 (13.9)	**<0.001**	Tobacco use† (%)	153 (4.1)	195 (9.6)	348 (6.0)	**<0.001**
Low physical activity‡ (%)	803 (17.5)	442 (18.2)	1245 (17.7)	**<0.001**	Low physical activity‡ (%)	1580 (49.8)	631 (33.8)	2211 (43.8)	**<0.001**
Waist circumference (cm; SD)	85.2 (11.6)	88.3 (11.1)	86.3 (11.5)	**<0.001**	Waist circumference (cm; SD)	90.4 (11.8)	92.9 (11.9)	91.3 (11.9)	**<0.001**
BMI (kg/m2; SD)	26.8 (4.9)	25.2 (4.1)	26.2 (4.6)	**<0.001**	BMI (kg/m2; SD)	27.7 (5.2)	26.2 (4.6)	27.2 (5.07)	**<0.001**
WHR	0.86 (0.08)	0.93 (0.09)	0.88 (0.09)	**<0.001**	WHR	0.89 (0.08)	0.95 (0.08)	0.91 (0.08)	**<0.001**
Relative grip strength§	0.36 (0.13)	0.50 (0.16)	0.41 (0.16)	**<0.001**	Relative grip strength§	0.30 (0.10)	0.44 (0.14)	0.34 (0.13)	**<0.001**
SBP (mmHg; SD)	127.3 (22.3)	131.0 (20.9)	128.6 (21.9)	**<0.001**	SBP (mmHg; SD)	126.8 (20.3)	132.2 (19.0)	128.6 (20.0)	**<0.001**
DBP (mmHg; SD)	80.4 (12.6)	81.2 (12.9)	80.7 (12.7)	**0.018**	DBP (mmHg; SD)	78.2 (10.8)	80.6 (11.4)	79.00 (11.1)	**<0.001**
Glucose (mg/dL; SD)	86.8 (30.5)	86.7 (25.9)	86.7 (28.7)	0.901	Glucose (mg/dL; SD)	100.7 (33.6)	100.9 (35.3)	100.7 (34.1)	0.929
Total cholesterol (mg/dL; SD)	204 (46.1)	196.3 (45.9)	200.8 (46.1)	**<0.001**	Total cholesterol (mg/dL; SD)	206.2 (45.7)	197.3 (46.2)	203.47 (46.0)	**0.002**
HDL-c (mg/dL; SD)	44.0 (10.2)	40.9 (10.0)	42.7 (10.2)	**<0.001**	HDL-c (mg/dL; SD)	45.2 (9.5)	42.3 (9.5)	44.3 (9.6)	**<0.001**
LDL-c (mg/dL; SD)	128.0 (35.9)	122.1 (37.3)	125.6 (36.5)	**<0.001**	LDL-c (mg/dL; SD)	127.6 (42.1)	119.9 (43.5)	125.2 (42.7)	**0.005**
Triglycerides (mg/dL; SD)	176.4 (103.7)	195.0 (120.9)	184.0 (111.5)	**<0.001**	Triglycerides (mg/dL; SD)	170.2 (80.4)	176.2 (103.4)	172.09 (88.2)	0.280
Hypertension (%)	1869 (38.6)	977 (36.1)	2846 (37.7)	**0.037**	Hypertension (%)	2368 (51.5)	1210 (47.5)	3578 (50.1)	**0.001**
Diabetes (%)	286 (5.9)	138 (5.1)	424 (5.6)	0.162	Diabetes (%)	584 (12.7)	261 (10.2)	845 (11.8)	**0.002**

BMI: body mass index; DBP: diastolic blood pressure; HDL-c: high-density lipoprotein; LDL-c: low density lipoprotein; SBP: systolic blood pressure; SD: standard deviation; WHR: waist-hip ratio. *Alcohol consumption was defined has alcohol consumption at least once a year. †Tobacco use was defined as current or previous tobacco use. ‡Low physical activity was defined as less than 600 metabolic equivalent x minutes per week. §Relative grip strength was calculated by dividing the absolute handgrip strength by BMI
